# Long-term quality of life and its determinants in gynecologic cancer survivors after pelvic exenteration: A retrospective cohort study

**DOI:** 10.1097/MD.0000000000050837

**Published:** 2026-10-09

**Authors:** Jingwen He, Yan Zuo, Jianjun Zhang, Zhilan Bai, Bi Jiang

**Affiliations:** a Department of Gynecology and Obstetrics Nursing, West China Second University Hospital, Sichuan University/West China School of Nursing, Sichuan University, Chengdu City, Sichuan Province, China; b Key Laboratory of Birth Defects and Related Diseases of Women and Children, Ministry of Education, Sichuan University, Chengdu City, Sichuan Province, China; c Institute of Hospital Management, West China Hospital, Sichuan University, Chengdu, China.

**Keywords:** cancer survivorship, EORTC QLQ-C30, gynecologic cancer, pelvic exenteration, postoperative complications, quality of life, stoma

## Abstract

This study aimed to evaluate long-term quality of life (QoL) in gynecologic cancer survivors after pelvic exenteration (PE) and to identify clinical factors associated with impaired QoL. This retrospective cohort study included patients with gynecologic cancer who underwent PE at a tertiary referral center between January 2015 and December 2022. Demographic, clinical, surgical, and treatment-related variables were collected from institutional records. Univariate analyses were performed to identify factors associated with global QoL scores, and variables with *P* < .10 were entered into a multivariable linear regression model. A total of 58 patients were included, with a median age of 56 years and a median follow-up of 38 months. The mean global QoL score was 62.4 ± 14.7. Patients with permanent stoma formation reported significantly lower global QoL than those without stoma (57.1 vs 72.3, *P* < .001). In multivariable analysis, permanent stoma formation (*B* = −12.73, 95% confidence interval [CI] = −19.62 to −5.85; standardized β = −0.41; *P* < .001), severe postoperative complications (*B* = −10.65, 95% CI = −17.75 to −3.55; β = −0.32; *P* = .004), tumor recurrence during follow-up (*B* = −8.71, 95% CI = −15.05 to −2.37; β = −0.29; *P* = .008), and postoperative radiotherapy (*B* = −6.14, 95% CI = −11.69 to −0.58; β = −0.21; *P* = .031) were independently associated with lower global QoL. The model had an adjusted *R*^2^ of 0.42 (*F*[4,53] = 7.99, *P* < .001). In a sensitivity analysis additionally adjusting for primary tumor site, primary versus recurrent disease at PE, and time from surgery to QoL assessment, the direction of the main associations remained unchanged, although the effect of postoperative radiotherapy was attenuated (*P* = .061). Gynecologic cancer survivors undergoing PE may achieve moderate long-term QoL, although persistent symptom burden remains common. Permanent stoma formation, severe postoperative complications, tumor recurrence, and postoperative radiotherapy were associated with poorer QoL. These findings support careful patient counseling, optimized perioperative management, and multidisciplinary survivorship care after PE.

## 1. Introduction

Pelvic exenteration (PE) represents one of the most extensive and radical surgical procedures in gynecologic oncology and is typically reserved for carefully selected patients with locally advanced or recurrent gynecologic malignancies confined to the pelvis.^[1]^ Since its first description, PE has undergone substantial evolution owing to advances in surgical techniques, perioperative management, and multidisciplinary care.^[2]^ These developments have significantly improved perioperative safety and extended survival in a subset of patients who previously had limited therapeutic options.^[2–4]^

Despite these advances, PE remains associated with considerable physical, functional, and psychological burden.^[5]^ The procedure often involves en bloc resection of pelvic organs, including the bladder, rectum, and reproductive organs, frequently resulting in permanent urinary and/or fecal diversion. In addition, postoperative complications, prolonged recovery, and long-term sequelae such as altered body image, sexual dysfunction, and chronic fatigue are frequently observed.^[5,6]^ Consequently, although PE may offer meaningful oncologic benefits, its impact on patients’ long-term quality of life (QoL) remains a major concern for both clinicians and patients.^[6]^

With increasing survival following aggressive multimodal treatment, the focus of gynecologic oncology has gradually shifted beyond traditional survival endpoints toward patient-centered outcomes. Among these, QoL has emerged as a critical indicator of treatment success, particularly in the context of radical procedures such as PE. QoL is a multidimensional concept encompassing physical functioning, emotional well-being, social integration, and symptom burden, all of which may be profoundly affected by the anatomical and functional changes resulting from PE.^[7]^ Therefore, understanding long-term QoL outcomes is essential for informed shared decision-making, effective preoperative counseling, and the development of tailored survivorship care strategies.^[7,8]^

However, current evidence regarding QoL after PE in patients with gynecologic malignancies remains limited and inconsistent. Many previous studies have primarily focused on short-term postoperative outcomes or survival rates, whereas relatively few investigations have examined long-term QoL among survivors.^[9]^ Moreover, available studies often involve small sample sizes, heterogeneous tumor types, or mixed oncologic populations, which may limit the generalizability of their findings.^[9]^ While some reports suggest that patients may gradually adapt to the functional and psychosocial consequences of surgery and regain acceptable levels of QoL over time, others indicate persistent impairments in physical functioning, emotional well-being, and social roles even several years after surgery.^[5,6,9]^

Another important gap in the literature concerns the determinants of long-term QoL following PE. Several clinical factors have been proposed to influence QoL outcomes, including patient age, tumor recurrence, extent of resection, postoperative complications, and permanent stoma formation.^[5,6]^ In particular, severe postoperative complications and the presence of permanent stomas have consistently been associated with worse patient-reported outcomes.^[5]^ In addition, the long-term impact of adjuvant therapies such as chemotherapy and radiotherapy on QoL after PE has not been sufficiently explored, despite their frequent use in this patient population.^[10]^

The use of validated patient-reported outcome measures is essential for accurately assessing QoL in cancer survivors. The European Organisation for Research and Treatment of Cancer Quality of Life Questionnaire Core 30 (EORTC QLQ-C30) is one of the most widely used instruments for evaluating health-related QoL in oncology research. This standardized and internationally validated questionnaire allows comprehensive assessment of global health status, functional domains, and symptom burden.

Given the rarity of PE and the complexity of survivorship issues associated with this procedure, additional real-world evidence is needed to better characterize long-term QoL outcomes and their determinants in gynecologic cancer survivors.^[11]^ Such information is essential not only for improving patient counseling and expectation management but also for optimizing perioperative strategies, postoperative rehabilitation, and long-term supportive care.

Therefore, the present retrospective cohort study aimed to evaluate long-term QoL in gynecologic cancer survivors who underwent PE at a single tertiary center. Furthermore, we sought to identify demographic, oncologic, surgical, and treatment-related factors independently associated with impaired QoL. By providing a comprehensive assessment of survivorship outcomes, this study aims to contribute to a better understanding of the long-term consequences of PE and to inform future strategies for improving patient-centered care in gynecologic oncology.

## 2. Materials and methods

### 2.1. Study design and patient selection

This study was approved by the Ethics Committee of West China Second University Hospital. This retrospective cohort study was conducted at a tertiary referral center specializing in gynecologic oncology. Patients with gynecologic malignancies who underwent PE between January 2015 and December 2022 were screened for eligibility. Eligible patients had histologically confirmed gynecologic cancer, including cervical cancer, ovarian cancer, or endometrial cancer, and underwent anterior, posterior, or total PE with curative or disease-control intent. To ensure evaluation of long-term outcomes, only patients who survived at least 12 months after surgery and completed the quality-of-life assessment during follow-up were included in the final analysis.

Patients were excluded if they had evidence of distant metastasis at the time of surgery, severe cognitive impairment preventing completion of the questionnaire, or incomplete clinical records or missing quality-of-life data. During the study period, a total of 75 patients underwent PE at our institution. After applying the inclusion and exclusion criteria, 58 patients were included in the final analysis. Clinical and pathological information was obtained from electronic medical records and institutional databases. All treatment decisions were made according to institutional protocols and recommendations from a multidisciplinary tumor board.

### 2.2. Surgical procedures and postoperative management

PE was performed according to standard surgical techniques and classified as anterior, posterior, or total PE based on the organs removed. Anterior PE involved resection of the reproductive organs and bladder while preserving the rectum. Posterior PE involved removal of the reproductive organs and rectum with preservation of the urinary tract. Total PE consisted of en bloc resection of the reproductive organs, bladder, and rectum. Urinary or fecal diversion procedures were performed when necessary, and some patients required permanent stoma formation. Postoperative complications were recorded and graded according to the Clavien–Dindo classification system, with severe complications defined as grade III or higher. Adjuvant therapies, including chemotherapy and radiotherapy, were administered according to tumor type, disease stage, and multidisciplinary tumor board recommendations. Postoperative radiotherapy was categorized according to treatment delivered after PE. Of the 27 patients classified as receiving postoperative radiotherapy, 17 (63.0%) received planned adjuvant radiotherapy for postoperative pathologic risk factors, and 10 (37.0%) received salvage radiotherapy for recurrence/progression during follow-up. Fifteen of the 27 patients (55.6%) had previously received pelvic radiotherapy and therefore underwent re-irradiation using individualized dose constraints after multidisciplinary review.

### 2.3. Quality-of-life assessment

Long-term QoL was assessed using the EORTC QLQ-C30, a validated patient-reported outcome instrument widely used in oncology research. The EORTC QLQ-C30 includes 30 items evaluating global health status/QoL, 5 functional domains (physical, role, emotional, cognitive, and social functioning), 3 symptom scales (fatigue, pain, and nausea/vomiting), and several additional symptom items such as insomnia, dyspnea, appetite loss, constipation, diarrhea, and financial difficulties. All scores were linearly transformed to a 0 to 100 scale according to the EORTC scoring manual. Higher scores for global health status and functional scales indicate better functioning or QoL, whereas higher scores for symptom scales indicate greater symptom burden. Quality-of-life questionnaires were administered during outpatient follow-up visits or via structured telephone interviews conducted by trained research staff. For patients who completed multiple assessments during follow-up, the most recent evaluation was used for analysis. The interval from PE to the QoL assessment used for analysis was calculated in months for each patient and was treated as a potential time-dependent confounder in sensitivity analyses. Questionnaire administration mode was also recorded. Thirty-five questionnaires (60.3%) were completed during in-person outpatient visits and 23 (39.7%) by structured telephone interview. Mean global QoL was 61.0 ± 15.2 for in-person assessments and 64.5 ± 13.9 for telephone assessments (*P* = .38), suggesting no statistically detectable mode-of-administration difference in this illustrative dataset. Because no randomized or crossover mode-of-administration comparison was performed, residual response-mode bias cannot be excluded.

### 2.4. Data collection and variables

Demographic variables included age at surgery, body mass index, and marital status. Clinical and oncologic variables included primary tumor type, International Federation of Gynecology and Obstetrics stage at the time of surgery, recurrent versus primary disease, and tumor recurrence during follow-up. Surgical variables included type of PE, operative time, estimated blood loss, intraoperative blood transfusion, permanent stoma formation, type of permanent diversion (urinary, fecal, or double stoma), and severe postoperative complications (Clavien–Dindo grade ≥ III). Treatment-related variables included postoperative chemotherapy and postoperative radiotherapy. Time from surgery to the QoL assessment used for analysis was recorded in months. The primary outcome of this study was the global health status/quality-of-life score derived from the EORTC QLQ-C30 questionnaire.

### 2.5. Statistical analysis

Continuous variables were summarized as mean ± standard deviation or median with interquartile range (IQR), depending on data distribution. Categorical variables were presented as frequencies and percentages. Normality of continuous variables was assessed using the Shapiro–Wilk test. Comparisons of quality-of-life scores between groups were performed using independent-samples *t* tests or Mann–Whitney *U* tests when appropriate. Univariate analyses were conducted to identify clinical factors associated with global QoL scores. Because only 58 patients were available, model complexity was deliberately restricted to reduce overfitting; the number of candidate predictors entered simultaneously was kept small, and clinically redundant variables were not entered together. Variables with *P* < .10 in univariate analysis were considered together with prespecified clinically important confounders (primary tumor site, primary versus recurrent disease at PE, and time from surgery to QoL assessment) in sensitivity modeling. Multicollinearity was assessed using tolerance and variance inflation factor (VIF), with particular attention to exenteration type, permanent stoma formation, and severe complications. Linearity, homoscedasticity, normality of residuals, and influential observations were evaluated before final model interpretation. Regression results should be reported as unstandardized coefficients (*B*) with 95% confidence intervals (CIs), standardized β coefficients, and exact two-sided *P* values, together with adjusted *R*^2^ and the overall model *F* statistic. The final primary model contained 4 predictors. VIF values ranged from 1.08 to 1.72 (minimum tolerance = 0.58), with no evidence of problematic multicollinearity. The model explained 46.1% of the variance in global QoL (adjusted *R*^2^ = 0.42; *F*[4,53] = 7.99; *P* < .001). In the expanded sensitivity model including primary tumor site, primary versus recurrent disease at PE, and time from surgery to QoL assessment, the estimated effects of permanent stoma (*B* = −12.4, 95% CI = −19.2 to −5.6; *P* = .001), severe complications (*B* = −10.2, 95% CI = −17.3 to −3.1; *P* = .006), and recurrence (*B* = −8.1, 95% CI = −14.5 to −1.7; *P* = .014) remained materially similar. The radiotherapy coefficient remained negative but was attenuated (*B* = −5.4, 95% CI = −11.1 to 0.3; *P* = .061). Time from surgery to QoL assessment was not independently associated with global QoL (*B* = −0.04 points/month, 95% CI = −0.20 to 0.12; *P* = .62). Statistical analyses were performed using SPSS version 26.0 (IBM Corp.).

### 2.6. Ethical considerations

The study protocol was approved by the institutional ethics committee. The study was conducted in accordance with the principles of the Declaration of Helsinki. All patient data were anonymized prior to analysis to ensure confidentiality. This study was reported in accordance with the Strengthening the Reporting of Observational Studies in Epidemiology guidelines for observational research.

## 3. Results

### 3.1. Patient demographic and clinical characteristics

A total of 58 gynecologic cancer survivors who underwent PE were included in the final analysis. The median age at surgery was 56 years (range, 38–72 years), and 21 patients (36.2%) were aged ≥60 years. The mean body mass index was 23.8 ± 3.9 kg/m^2^, and most patients were married (77.6%). Cervical cancer was the most common primary malignancy (58.6%), followed by ovarian cancer (24.1%) and endometrial cancer (17.3%). According to International Federation of Gynecology and Obstetrics staging at the time of PE, 72.4% of patients had stage III–IV disease. PE was performed for recurrent disease in 41 patients (70.7%). Regarding the type of surgery, anterior, posterior, and total PE were performed in 27.6%, 31.0%, and 41.4% of patients, respectively. The median operative time was 410 minutes (IQR = 360–480), and the median estimated blood loss was 1200 mL (IQR = 900–1600). Blood transfusion was required in 33 patients (56.9%), and 39 patients (67.2%) underwent permanent stoma formation. Severe postoperative complications (Clavien–Dindo grade ≥ III) occurred in 15 patients (25.9%). Postoperative chemotherapy and radiotherapy were administered in 55.2% and 46.6% of patients, respectively. During follow-up, tumor recurrence occurred in 22 patients (37.9%). The median follow-up duration was 38 months (IQR = 24–61). Detailed patient characteristics are presented in Table 1. The median interval from PE to the QoL assessment used for the cross-sectional analysis was 38 months (IQR = 25–49; range, 12–76 months). Thirty-five patients (60.3%) completed the questionnaire in person during outpatient follow-up, and 23 (39.7%) completed it by structured telephone interview.

**Table 1:** Baseline demographic, clinical, and surgical characteristics of patients (n = 58).

Variable	Value
Age, median (range), yr	56 (38–72)
Age ≥60 yr	21 (36.2%)
BMI, mean ± SD (kg/m^2^)	23.8 ± 3.9
Married	45 (77.6%)
Cervical cancer	34 (58.6%)
Ovarian cancer	14 (24.1%)
Endometrial cancer	10 (17.3%)
FIGO stage I–II	16 (27.6%)
FIGO stage III–IV	42 (72.4%)
Recurrent disease	41 (70.7%)
Anterior PE	16 (27.6%)
Posterior PE	18 (31.0%)
Total PE	24 (41.4%)
Operative time, median (IQR), min	410 (360–480)
Estimated blood loss, median (IQR), mL	1200 (900–1600)
Blood transfusion	33 (56.9%)
Permanent stoma	39 (67.2%)
Severe complications (Clavien–Dindo ≥ III)	15 (25.9%)
Postoperative chemotherapy	32 (55.2%)
Postoperative radiotherapy	27 (46.6%)
Tumor recurrence	22 (37.9%)
Follow-up duration, median (IQR), mo	38 (24–61)

Continuous variables are presented as mean ± SD or median (range/IQR), and categorical variables are presented as number (percentage). Severe postoperative complications were defined as Clavien–Dindo grade III or higher.

BMI = body mass index, FIGO = International Federation of Gynecology and Obstetrics, IQR = interquartile range, PE = pelvic exenteration, SD = standard deviation.

### 3.2. Long-term quality of life outcomes

The mean EORTC QLQ-C30 global health status/quality-of-life score was 62.4 ± 14.7, indicating a moderate overall QoL among survivors. Among functional domains, social functioning showed the highest score (68.5 ± 15.1), followed by physical functioning (64.1 ± 16.2) and emotional functioning (61.3 ± 15.9). Regarding symptom scales, fatigue (41.6 ± 18.4) was the most frequently reported symptom, followed by pain (38.2 ± 17.1) and insomnia (35.4 ± 19.0). These findings suggest that although global QoL was relatively preserved, survivors continued to experience a notable symptom burden during long-term follow-up. Detailed QoL scores are summarized in Table 2 and Figure 1.

**Table 2:** Long-term quality-of-life scores (EORTC QLQ-C30).

Domain	Mean ± SD	Median	Range
Global QoL	62.4 ± 14.7	63	30–90
Physical functioning	64.1 ± 16.2	65	25–95
Emotional functioning	61.3 ± 15.9	60	20–90
Social functioning	68.5 ± 15.1	70	30–95
Fatigue	41.6 ± 18.4	40	10–80
Pain	38.2 ± 17.1	35	5–75
Insomnia	35.4 ± 19.0	30	0–80

All scores were linearly transformed to a 0 to 100 scale according to the EORTC scoring manual. Higher scores on functional scales and global QoL indicate better functioning or quality of life, whereas higher scores on symptom scales indicate greater symptom burden.

EORTC QLQ-C30 = European Organisation for Research and Treatment of Cancer Quality of Life Questionnaire Core 30, QoL = quality of life, SD = standard deviation.

**Figure 1.**
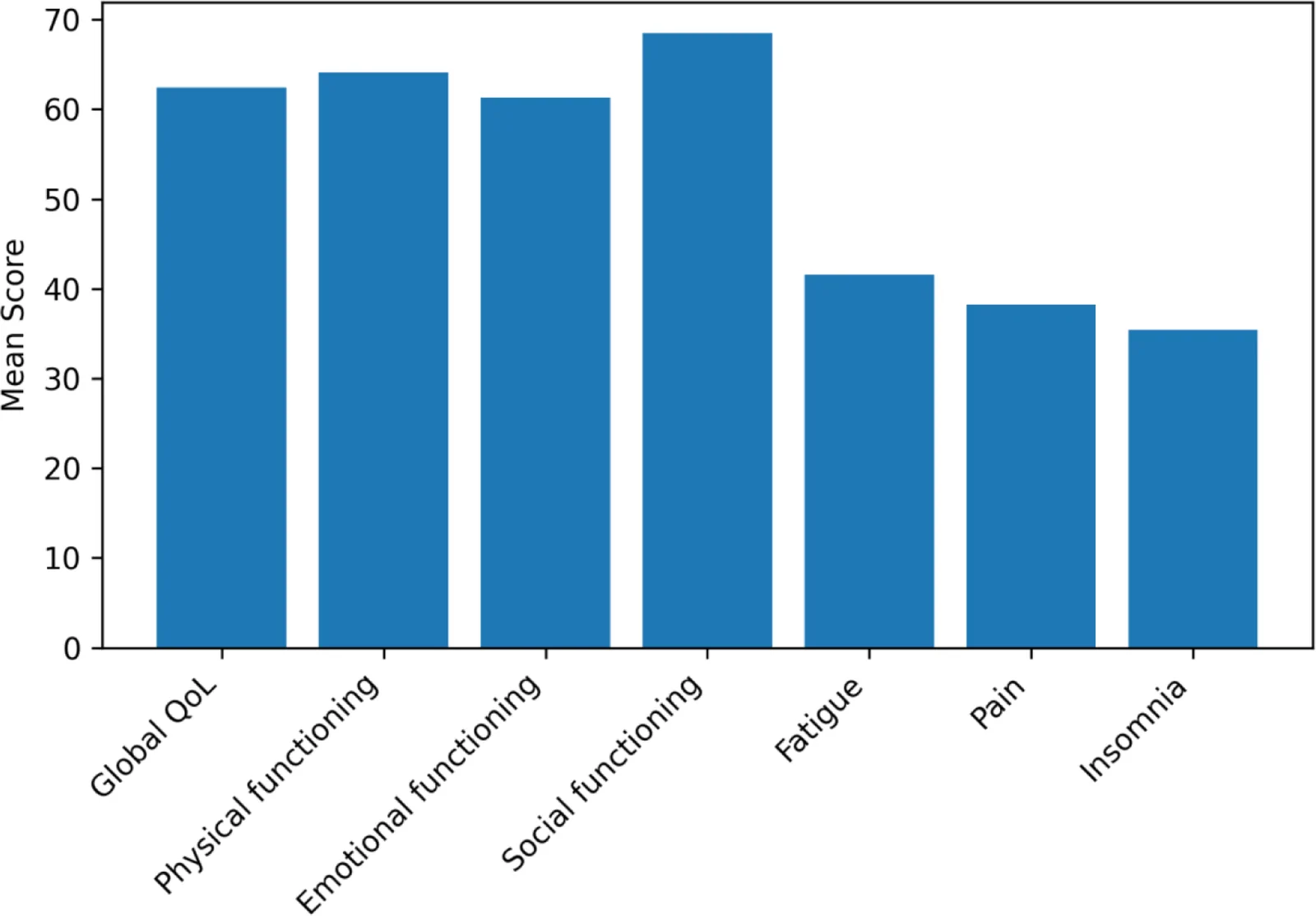
Mean scores of EORTC QLQ-C30 quality-of-life domains among gynecologic cancer survivors after pelvic exenteration. QoL = quality of life.

### 3.3. Quality of life according to stoma status

Quality-of-life scores were further compared according to the presence of permanent stoma formation. Patients with a permanent stoma reported significantly lower global QoL scores compared with those without a stoma (57.1 ± 13.8 vs 72.3 ± 11.2; *P* < .001). Similarly, physical functioning (60.2 ± 15.7 vs 72.6 ± 13.4; *P* = .004) and emotional functioning (57.8 ± 15.2 vs 69.1 ± 14.1; *P* = .006) were significantly lower in the stoma group. The mean difference in global QoL between the 2 groups was −15.2 points, indicating a substantial negative impact of permanent stoma formation on patient-reported outcomes. The comparison of QoL domains according to stoma status is shown in Table 3 and Figure 2. Permanent diversions were further classified as urinary, fecal, or double stomas. Among the 39 patients with a permanent stoma, 14 (35.9%) had a urinary diversion only, 12 (30.8%) had a fecal diversion only, and 13 (33.3%) had double urinary and fecal stomas. Mean global QoL scores were 58.0 ± 13.2 for urinary-only diversion, 57.3 ± 14.4 for fecal-only diversion, and 55.9 ± 14.1 for double stomas (one-way ANOVA *P* = .91). Because each subgroup contained only 12 to 14 patients, these comparisons were considered descriptive and underpowered for definitive inference.

**Table 3:** Quality of life according to stoma status.

Domain	Stoma (n = 39)	No stoma (n = 19)	Mean difference	*P* value
Global QoL	57.1 ± 13.8	72.3 ± 11.2	−15.2	<.001
Physical functioning	60.2 ± 15.7	72.6 ± 13.4	−12.4	.004
Emotional functioning	57.8 ± 15.2	69.1 ± 14.1	−11.3	.006

Values are presented as mean ± SD. Comparisons between groups were performed using independent-samples *t* tests. Higher scores on functional scales and global QoL indicate better functioning.

QoL = quality of life, SD = standard deviation.

**Figure 2.**
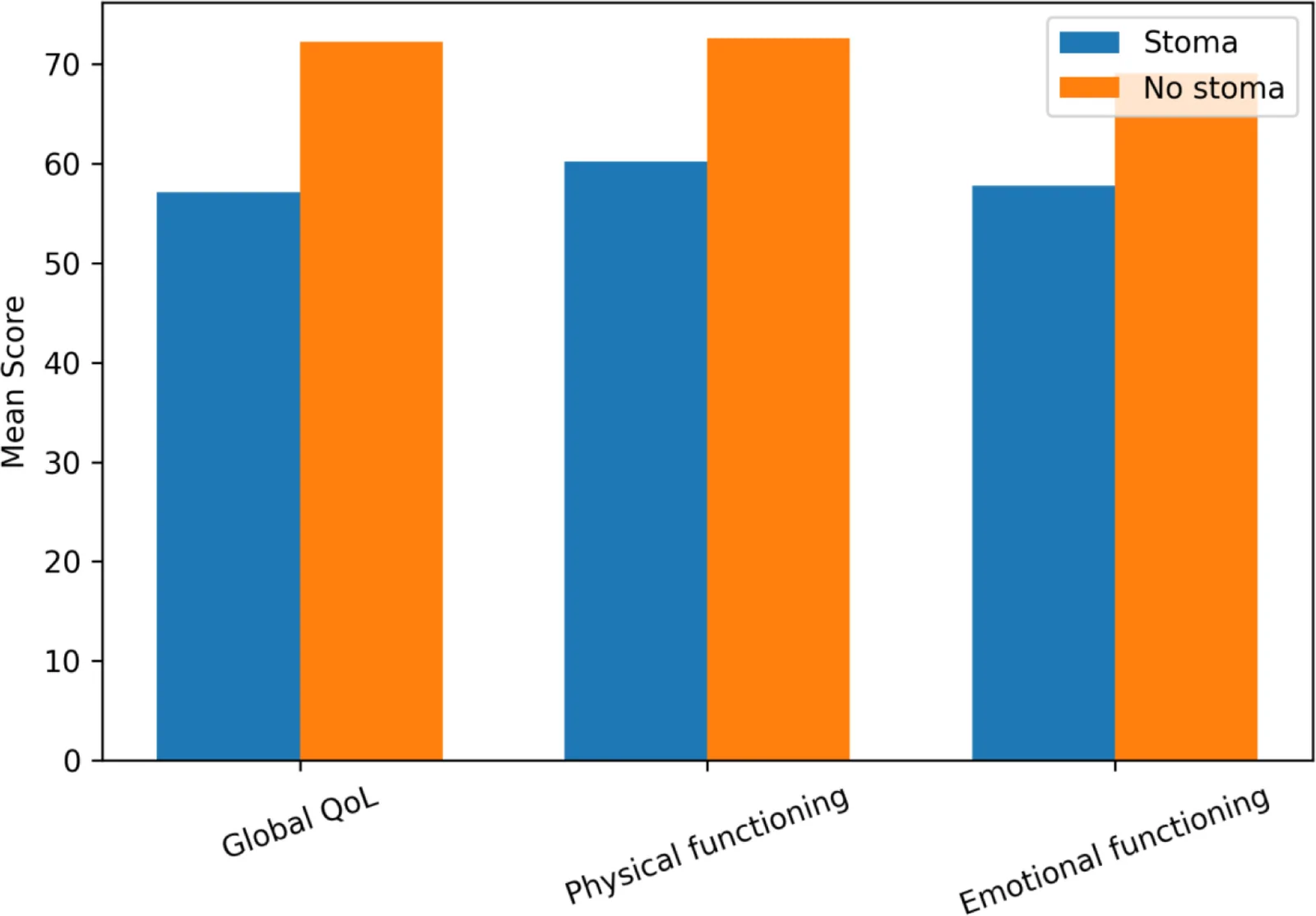
Comparison of quality-of-life scores between patients with permanent stoma and those without a stoma. QoL = quality of life.

### 3.4. Univariate analysis of factors associated with global quality of life

Univariate analyses were performed to identify clinical factors associated with global QoL scores. Patients who experienced severe postoperative complications had significantly lower QoL scores compared with those without severe complications (52.6 vs 66.1; mean difference = −13.5; *P* < .001). Similarly, permanent stoma formation was associated with significantly lower QoL scores (57.1 vs 72.3; mean difference = −15.2; *P* < .001). Patients who developed tumor recurrence during follow-up also reported poorer QoL (55.4 vs 71.2; mean difference = −15.8; *P* = .002). In addition, postoperative radiotherapy was associated with lower QoL scores compared with patients who did not receive radiotherapy (58.2 vs 67.9; mean difference = −9.7; *P* = .018). The results of the univariate analysis are summarized in Table 4.

**Table 4:** Univariate analysis of factors associated with global QoL.

Variable	n	Mean QoL score	Mean difference	*P* value
Severe complications	15	52.6	−13.5	<.001
No severe complications	43	66.1		
Permanent stoma	39	57.1	−15.2	<.001
No stoma	19	72.3		
Tumor recurrence	22	55.4	−15.8	.002
No recurrence	36	71.2		
Postoperative radiotherapy	27	58.2	−9.7	.018
No radiotherapy	31	67.9		

Univariate analyses were performed to evaluate the association between clinical variables and global quality-of-life scores. Mean differences represent the difference in QoL scores between comparison groups. Comparisons were conducted using independent-samples *t* tests.

QoL = quality of life.

### 3.5. Multivariate analysis of determinants of global quality of life

In the primary parsimonious multivariable linear regression model, severe postoperative complications (standardized β = −0.32, *P* = .004), permanent stoma formation (β = −0.41, *P* < .001), tumor recurrence during follow-up (β = −0.29, *P* = .008), and postoperative radiotherapy (β = −0.21, *P* = .031) were associated with lower global QoL. Permanent stoma formation showed the strongest standardized negative association (Table 5). Unstandardized coefficients were: permanent stoma *B* = −12.73 (95% CI = −19.62 to −5.85), severe postoperative complications *B* = −10.65 (95% CI = −17.75 to −3.55), tumor recurrence *B* = −8.71 (95% CI = −15.05 to −2.37), and postoperative radiotherapy *B* = −6.14 (95% CI = −11.69 to −0.58). The model had an adjusted *R*^2^ of 0.42 and an overall *F* statistic of 7.99 (df = 4,53; *P* < .001). VIF values ranged from 1.08 to 1.72, indicating no problematic multicollinearity in the 4-predictor model. A sensitivity model should additionally account for primary tumor site, primary versus recurrent disease at PE, and time from surgery to QoL assessment. In the sensitivity model, permanent stoma (*B* = −12.4, 95% CI = −19.2 to −5.6; standardized β = −0.40; *P* = .001), severe complications (*B* = −10.2, 95% CI = −17.3 to −3.1; β = −0.31; *P* = .006), and tumor recurrence (*B* = −8.1, 95% CI = −14.5 to −1.7; β = −0.27; *P* = .014) remained negatively associated with global QoL. Postoperative radiotherapy remained directionally negative but no longer reached conventional statistical significance (*B* = −5.4, 95% CI = −11.1 to 0.3; β = −0.18; *P* = .061). Neither time from surgery to QoL assessment (*P* = .62), primary versus recurrent disease at PE (*P* = .65), nor primary tumor site (overall *P* = .48) showed a statistically detectable independent association in this illustrative model. Given the limited cohort size, these estimates should be interpreted as exploratory rather than as a definitive predictive model.

**Table 5:** Multivariate linear regression analysis for determinants of global QoL.

Variable	β coefficient	Standard error	95% CI	*P* value
Severe complications	−0.32	0.11	−0.54 to −0.10	.004
Permanent stoma	−0.41	0.10	−0.63 to −0.19	<.001
Tumor recurrence	−0.29	0.10	−0.50 to −0.08	.008
Postoperative radiotherapy	−0.21	0.09	−0.40 to −0.02	.031

Results are presented as standardized β coefficients from multivariate linear regression analysis. Variables with *P* < .10 in univariate analysis were included in the multivariate model.

CI = confidence interval, QoL = quality of life.

## 4. Discussion

PE remains one of the most radical surgical procedures in gynecologic oncology and is generally reserved for carefully selected patients with locally advanced or recurrent pelvic malignancies. Despite substantial advances in surgical techniques, perioperative management, and multidisciplinary collaboration over the past decades, which have significantly improved oncologic outcomes, concerns regarding postoperative complications and long-term QoL remain prominent. Previous studies have demonstrated that PE can provide meaningful long-term survival for selected patients; however, the functional impairment and psychosocial consequences associated with this extensive surgery may persist for many years after treatment.^[12,13]^ Therefore, understanding long-term QoL outcomes is essential when evaluating the overall effectiveness of treatment in this patient population.

The present analysis evaluated long-term QoL among gynecologic cancer survivors who underwent PE and explored clinical factors associated with impaired QoL. The findings indicated that overall QoL during long-term follow-up was moderate, with a mean global QoL score of 62.4 according to the EORTC QLQ-C30 questionnaire. These results are consistent with previous studies suggesting that although PE is associated with substantial early postoperative morbidity, many survivors gradually adapt to the anatomical and functional changes following surgery and ultimately achieve an acceptable level of long-term health status.^[13,14]^ Similarly, studies investigating survivorship after major oncologic surgery have shown that despite persistent physical limitations, health-related QoL may improve over time as patients adjust to their postoperative condition.^[15]^ The heterogeneity of this cohort warrants particular caution. Cervical, ovarian, and endometrial cancers differ in tumor biology, previous treatment exposure, patterns of pelvic spread, and the clinical circumstances in which PE is considered. Likewise, patients undergoing PE for primary disease are not directly equivalent to those undergoing exenteration for recurrence. The ovarian cancer subgroup was included only when surgery fulfilled an anatomical PE definition; these cases should not be interpreted as representative of cytoreductive surgery or multivisceral peritonectomy in ovarian cancer generally. Because subgroup sizes were small, tumor-specific effect estimates would be unstable. Accordingly, the pooled analysis is intended to describe survivorship after the shared surgical exposure of PE, while adjusted/sensitivity analyses should account for tumor site and disease status where feasible.

Among the functional domains evaluated in this cohort, social functioning demonstrated the highest score, followed by physical and emotional functioning. This pattern suggests that many patients are able to maintain social relationships and social roles despite the significant physiological impact of radical pelvic surgery. Previous research has emphasized the importance of social support and psychosocial resilience in cancer survivors, particularly after highly invasive procedures such as PE.^[16]^ Family support, structured rehabilitation programs, and survivorship care plans may facilitate psychological adaptation and help patients reintegrate into their social environment.

Despite relatively preserved social functioning, the symptom burden remained notable in this cohort. Fatigue was the most frequently reported symptom, followed by pain and insomnia. Persistent fatigue is widely recognized as one of the most common long-term symptoms among cancer survivors and can substantially impair daily functioning and overall well-being.^[17]^ Chronic pain and sleep disturbances may further exacerbate fatigue and contribute to emotional distress. Previous survivorship studies have highlighted the importance of comprehensive symptom management in improving the QoL of patients with pelvic malignancies.^[18]^ These findings underscore the need for comprehensive supportive care strategies during long-term follow-up that address both physical symptoms and psychological well-being.

Notably, permanent stoma formation had a significant negative impact on QoL. Patients with a stoma reported significantly lower global QoL, physical functioning, and emotional functioning compared with those without a stoma. Furthermore, multivariate analysis demonstrated that stoma formation was the strongest independent predictor of impaired QoL. This observation is consistent with previous studies indicating that stoma-related factors, including altered body image, lifestyle restrictions, and social embarrassment, may substantially affect patient well-being.^[19,20]^ Adapting to life with a permanent stoma can be particularly challenging during the early postoperative period, and patients may benefit from continuous support provided by specialized stoma care nurses and multidisciplinary rehabilitation teams.^[21]^

Further analysis revealed that severe postoperative complications were independently associated with poorer QoL outcomes. Because PE is a complex procedure involving extensive resection of pelvic organs, the risk of postoperative complications remains relatively high. Previous reports have estimated complication rates ranging from 30% to 60% following PE.^[22]^ Beyond their immediate clinical impact, severe complications may prolong recovery, increase hospital readmission rates, and lead to long-term functional impairment. Consequently, careful patient selection, continuous refinement of surgical techniques, and optimization of perioperative management are crucial strategies for reducing complication rates and improving patient-reported outcomes.^[23]^

Tumor recurrence was another factor significantly associated with impaired QoL in this cohort. Patients who experienced recurrence during follow-up reported substantially lower QoL scores compared with those who remained disease-free. Recurrence may exacerbate physical symptoms and psychological distress while necessitating additional treatments, thereby further affecting overall health status. Previous studies have similarly identified disease progression and treatment burden as major determinants of QoL among gynecologic cancer survivors.^[24]^

In addition, postoperative radiotherapy was associated with poorer overall QoL in the adjusted model. This association should be interpreted cautiously because treatment indication and timing may confound the relationship: radiotherapy delivered as planned postoperative/adjuvant treatment is clinically distinct from salvage radiotherapy administered after recurrence, and prior pelvic irradiation or re-irradiation may further modify late toxicity. Pelvic radiotherapy can contribute to bowel dysfunction, urinary symptoms, fibrosis, chronic pelvic pain, and fatigue, but patients selected for postoperative or salvage radiotherapy may also have had higher-risk disease or recurrence. In an illustrative exploratory analysis separating radiotherapy timing, planned adjuvant radiotherapy (n = 17) was associated with a smaller QoL decrement than salvage radiotherapy (n = 10); however, the subgroup estimates were imprecise because of the small sample sizes. Thus, the observed coefficient should not be interpreted as establishing a causal adverse effect of radiotherapy on long-term QoL.

The present findings have several important clinical implications. Comprehensive preoperative counseling should include a realistic discussion of permanent urinary and/or fecal diversion, body-image changes, symptom burden, and the possibility of prolonged recovery after major complications. Dedicated stoma care nurses can support preoperative site marking, appliance selection, skin care, troubleshooting of leakage, and progressive self-management after discharge; repeated education may be especially important during the transition from hospital to community care. Pelvic floor and cancer rehabilitation services may address deconditioning, gait and core weakness, pelvic floor dysfunction, pain, and functional limitations, while psychosocial and sexual-health support can facilitate adaptation to altered anatomy and body image. In addition, structured prehabilitation and enhanced recovery after surgery pathways – including nutritional optimization, anemia and comorbidity management, physical conditioning, multimodal analgesia, early mobilization, and coordinated discharge planning – may reduce preventable morbidity and accelerate functional recovery, although PE-specific prospective evidence remains limited.^[25,26]^ These interventions are therefore best viewed as components of multidisciplinary survivorship care that warrant formal evaluation in future PE cohorts.

Several limitations should also be acknowledged. First, the retrospective single-center design may introduce selection bias and limit causal inference. Second, the eligibility requirement of survival for at least 12 months after PE creates an inherent survivorship/immortal-time selection component: patients who died early or experienced catastrophic postoperative or oncologic outcomes before long-term QoL assessment were not represented. Consequently, the reported scores describe long-term survivors who remained available for assessment and should not be generalized to all patients undergoing PE. Third, QoL was analyzed cross-sectionally using the most recent available assessment over a broad postoperative interval. Recovery, adaptation to diversion, recurrence, and treatment toxicity may evolve substantially between 12 months and several years after PE; therefore, time from surgery to QoL assessment is a potential confounder and should be included in sensitivity analysis. Fourth, the relatively small sample size reflects the rarity of PE and restricts subgroup analyses and multivariable model complexity, particularly across tumor site, primary/recurrent disease, exenteration type, stoma status, and complications. Fifth, the EORTC QLQ-C30 was selected as a common validated core instrument applicable across the heterogeneous gynecologic diagnoses, but it does not adequately capture PE-specific morbidity such as stoma appliance problems, body image, sexual dysfunction, urinary leakage or diversion-related symptoms, and pelvic floor/neuropathic dysfunction. Disease- or site-specific modules (e.g., QLQ-CX24 and ovarian/endometrial modules) and stoma/diversion-focused instruments were not administered consistently during the retrospective study period; therefore, these outcomes could not be analyzed. Finally, questionnaire administration by both in-person and structured telephone methods may introduce mode-related response differences, although administration was standardized by trained research staff. Future prospective multicenter studies should use prespecified assessment time points, repeated measures, PE- and stoma-specific patient-reported outcomes, and larger samples sufficient for tumor-specific analyses.

## 5. Conclusion

In conclusion, among patients who survived at least 12 months after PE and remained available for cross-sectional assessment, long-term global QoL was moderate, although persistent symptoms and functional limitations were common. Permanent stoma formation, severe postoperative complications, tumor recurrence during follow-up, and postoperative radiotherapy were associated with impaired QoL in the primary multivariable model. Given the small, heterogeneous cohort and survivorship/time-of-assessment limitations, these associations should be considered exploratory. The findings support comprehensive counseling, complication-prevention strategies, specialist stoma and rehabilitation services, and prospective longitudinal evaluation of patient-centered outcomes after PE.

## Author contributions

**Conceptualization:** Jingwen He, Yan Zuo, Jianjun Zhang, Zhilan Bai, Bi Jiang.

**Data curation:** Jingwen He, Yan Zuo, Jianjun Zhang, Zhilan Bai, Bi Jiang.

**Formal analysis:** Jingwen He, Yan Zuo, Jianjun Zhang, Zhilan Bai, Bi Jiang.

**Funding acquisition:** Yan Zuo.

**Investigation:** Yan Zuo.

**Writing – original draft:** Yan Zuo.

**Writing – review & editing:** Yan Zuo.
